# Vocal cord dysfunction/inducible laryngeal obstruction—2022 Melbourne Roundtable Report

**DOI:** 10.1111/resp.14518

**Published:** 2023-05-23

**Authors:** Paul Leong, Peter G. Gibson, Anne E. Vertigan, Mark Hew, Vanessa M. McDonald, Philip G. Bardin

**Affiliations:** ^1^ Monash Health Melbourne Victoria Australia; ^2^ Monash University Melbourne Victoria Australia; ^3^ John Hunter Hospital Newcastle New South Wales Australia; ^4^ Centre of Excellence in Treatable Traits University of Newcastle Newcastle New South Wales Australia; ^5^ Alfred Hospital Melbourne Victoria Australia

**Keywords:** asthma, complex breathlessness, inducible laryngeal obstruction, paradoxical vocal fold motion, vocal cord dysfunction

## Abstract

Vocal cord dysfunction/inducible laryngeal obstruction (VCD/ILO), is a common condition characterized by breathlessness associated with inappropriate laryngeal narrowing. Important questions remain unresolved, and to improve collaboration and harmonization in the field, we convened an international Roundtable conference on VCD/ILO in Melbourne, Australia. The aims were to delineate a consistent approach to VCD/ILO diagnosis, appraise disease pathogenesis, outline current management and model(s) of care and identify key research questions. This report summarizes discussions, frames key questions and details recommendations. Participants discussed clinical, research and conceptual advances in the context of recent evidence. The condition presents in a heterogenous manner, and diagnosis is often delayed. Definitive diagnosis of VCD/ILO conventionally utilizes laryngoscopy demonstrating inspiratory vocal fold narrowing >50%. Computed tomography of the larynx is a new technology with potential for swift diagnosis that requires validation in clinical pathways. Disease pathogenesis and multimorbidity interactions are complex reflecting a multi‐factorial, complex condition, with no single overarching disease mechanism. Currently there is no evidence‐based standard of care since randomized trials for treatment are non‐existent. Recent multidisciplinary models of care need to be clearly articulated and prospectively investigated. Patient impact and healthcare utilization can be formidable but have largely escaped inquiry and patient perspectives have not been explored. Roundtable participants expressed optimism as collective understanding of this complex condition evolves. The Melbourne VCD/ILO Roundtable 2022 identified clear priorities and future directions for this impactful condition.

**Summary of key points**

**Diagnosis**

- VCD/ILO can present in many ways and the phenotype concept may be a promising way to develop and improve understanding.
- Laryngoscopy with provocation is the gold standard to verify abnormal laryngeal closure, but diagnostic algorithms for dynamic CT are promising and require testing.
- Developing rapid, readily available tests to rule in/out VCD/ILO should be a priority.

**Pathogenesis**

- Causal pathways are incompletely understood.
- Phenotyping may help elucidate causal pathways, which may vary in importance in different phenotypes and may need to be addressed individually.
- The effect of multimorbidities is complex, often bidirectional and incompletely understood.

**Treatment**

- Speech pathology is the mainstay first line treatment, but geographical access varies, and there are no randomized data.
- Treatment algorithms incorporating other options need to be developed and tested.
- Multidisciplinary models of care are optimal, and standards of care could be defined based on staff capability rather than minimum staffing.

## INTRODUCTION

Vocal cord dysfunction/inducible laryngeal obstruction (VCD/ILO) refers to breathlessness in association with inappropriate laryngeal closure. Clinical presentations are heterogenous, occurring in numerous and diverse clinical settings.^1^, ^2^, ^3^, ^4^, ^5^, ^6^, ^7^, ^8^, ^9^, ^10^ VCD/ILO can be a sole presenting disorder, a mimic of other disorders including seemingly refractory asthma or act as a multimorbidity with asthma or chronic obstructive pulmonary disease (COPD).^10^ The coexists in up to 50% of some diseases such as asthma^11^ and chronic cough,^11^ can have a profound clinical impact, and has been identified as a national and international clinical and research priority.^1^

The past two decades has witnessed a profusion of interest, and important advances. The neologism ILO was introduced,^12^ continuous laryngoscopy during exercise (CLE) has provided a wealth of data,^2^, ^13^ and exercise‐ILO prominently features in recent International Olympic Committee publications.^14^, ^15^ There is growing appreciation of anatomical^3^, ^16^, ^17^ and clinical^18^ phenotypes, and the complexity of laryngeal dysfunction.^19^ Relationships between severe asthma, VCD/ILO and dysfunctional breathing are beginning to be understood.^9^, ^20^ Multidisciplinary care is recognized as important globally^17^, ^21^, ^22^ and quantitative techniques for laryngeal narrowing have been developed.^23^, ^24^, ^25^ It is therefore exciting to critically review and to discuss approaches.

Recognition of VCD/ILO varies globally and there are no well‐accepted standards or pathways. Epidemiology remains uncertain outside of specific contexts and management is not standardized since there are no randomized data. Globally, models of care are not evidence‐based with considerable heterogeneity,^22^ funding for clinical services and research remains challenging and the vast majority of primary data have been generated in single centres. Finally, promising diagnostic paradigms and strategic initiatives including clinical and research collaborations, multidimensional assessments, online resources and patient perspectives are nascent.

The Australian National Health and Medical Research Council Centre of Excellence in Asthma Treatable Traits seeks to define precision medicine strategies for individuals with asthma, and within this research programme VCD/ILO has been identified as an important treatable trait.^26^ As an activity of the Centre's knowledge generation and research translation agenda, an international VCD/ILO Roundtable was convened in Melbourne, Australia on 23–24 June 2022 with 31 national and international delegates attending in hybrid format. A list of attendees and supplemental information can be found in the Supporting Information S1 and S2.

The Roundtable aimed to frame a consistent approach to VCD/ILO diagnosis with a focus on adults, explore pathogenesis and contributors, outline management and model(s) of care and delineate research questions. This report summarizes discussions, key clinical and questions and outlines pertinent recommendations.

## ASSESSMENT AND DIAGNOSIS

Case history remains a key component. The condition occurs intermittently and has been confused with asthma since symptoms overlap. Asthma and VCD/ILO frequently coexist.^9^, ^27^, ^28^ Some symptoms may be more indicative of VCD/ILO including breathlessness with a ‘tight throat’, difficulty with inhalation and voice changes or loss of voice during episodes of breathlessness. Cough is common and short acting beta‐agonists usually provide little or no relief. With VCD/ILO night symptoms are rare and if present are commonly associated with gastroesophageal reflux or poorly controlled coexistent asthma. Exercise‐ILO (EILO) occurs during exercise, often in young athletes. Finally, physical examination is seldom contributary although raucous ‘stridor’ inspiratory breath sounds may be suggestive of VCD/ILO.

Discussion was generated around VCD/ILO phenotypes as recently proposed.^18^ At least four distinct clinical phenotypes may be recognized:

- Classic VCD/ILO (e.g., Kent's seminal description^29^),
- Lung‐disease associated VCD/ILO,^7^, ^28^, ^30^
- Exercise‐ILO (EILO),^14^
- Incident‐associated VCD/ILO.^5^, ^6^, ^31^

Cough‐associated VCD/ILO probably represents another group.^11^, ^32^

Phenotyping was regarded as a promising approach to heterogeneity and complexity. Effective phenotyping should be simple, inclusive and recognizable. Phenotyping should facilitate appropriate diagnostic and management pathways: these now require urgent investigation and validation.

Definitive diagnosis of VCD/ILO is made by laryngoscopy, and the methodology outlined by Morris and Christopher^10^ remains the conventional confirmatory approach outside of exercise‐ILO. Typically, inspiratory vocal fold narrowing >50% is used for case definition and can be augmented by provocation if the initial examination is negative. Criteria for expiratory adduction are less well established. The presence of vocal fold narrowing accompanied by typical patient symptoms increases diagnostic certainty although many patients may have only mild symptoms during episodes of VCD/ILO visible on laryngoscopy.

The diagnosis of EILO is best made by exercise provocation using continuous laryngoscopy with exercise (CLE). In EILO, supraglottic narrowing is commonplace, and Maat and co‐workers have devised a grading of severity encompassing supraglottic and glottic dimensions^13^ with explicit diagnostic criteria elaborated.^1^, ^2^, ^13^

A recent Delphi study^33^ ascertained international expert opinion on the diagnosis of VCD/ILO and there was consensus that the diagnosis is best established based on accepted procedures and criteria as outlined in the Delphi; it is hoped this will facilitate and improve standardization of clinical and research activity.

**FIGURE 1:**
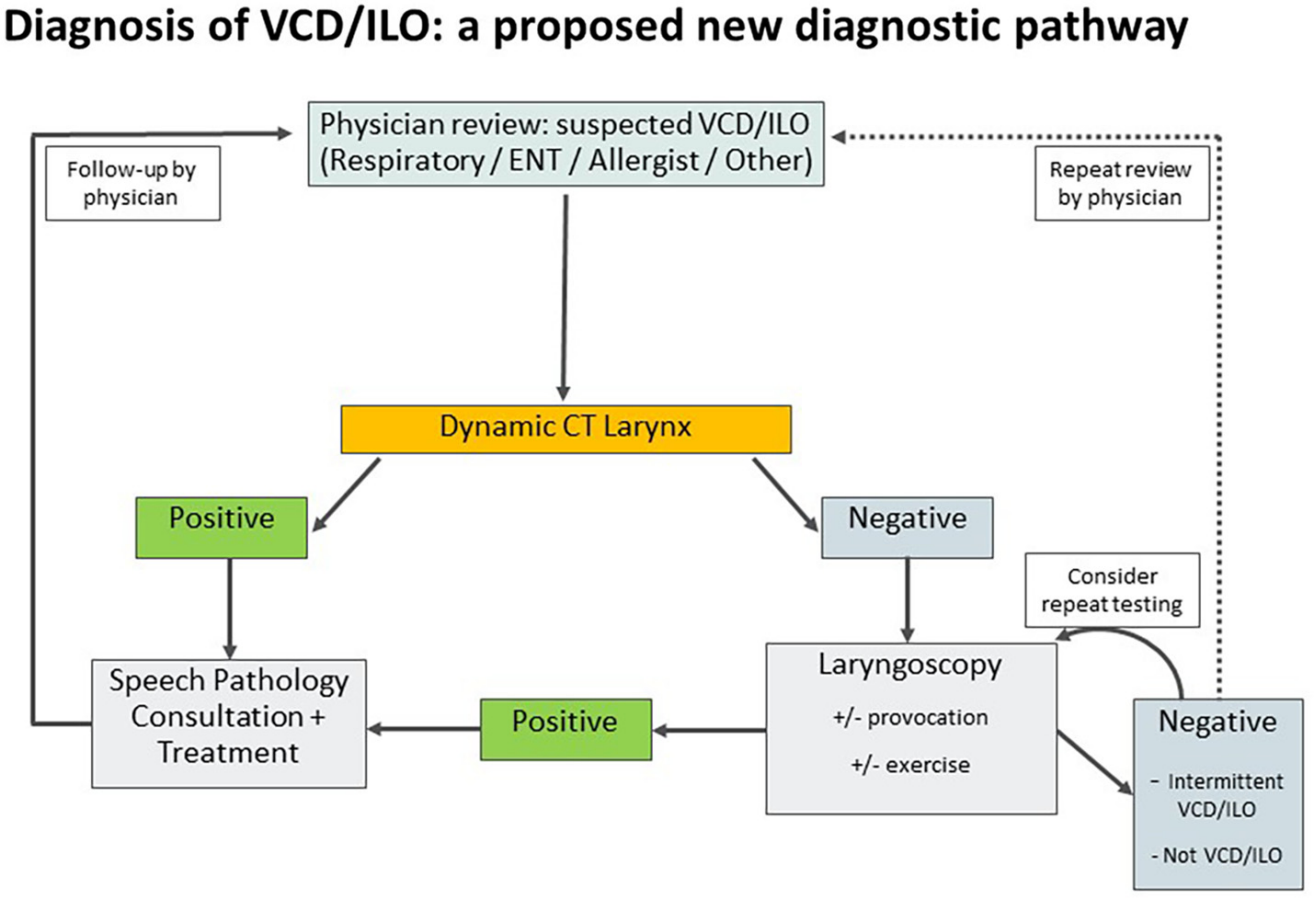
Proposed CT‐first diagnostic algorithm. Proposed diagnostic and management algorithm for VCD/ILO. Initial review by a specialist can be followed by CT larynx if the condition is considered as a diagnostic option. If CT imaging is diagnostic the patient can be directly referred for speech pathology review and management. Negative imaging requires review, preferably by a multidisciplinary team (MDT), and laryngoscopy (and provocation if indicated). Positive laryngoscopy can trigger referral for speech pathology but if negative results persist the patient can either have repeat laryngoscopy or have referral back to the initial specialist for consideration of other diagnoses. The new pathway ‘protects’ laryngoscopy providing potentially greater access to definitive diagnosis by laryngoscopy for complex cases. The pathway now requires testing and validation.

Laryngoscopy is dependent on expert procedural skills, can be unpleasant, is frequently unavailable at short notice when symptoms of VCD/ILO occur, and is rarely available outside metropolitan specialist clinics in high‐income countries. Other diagnostic modalities have been tried including spirometry,^34^ ultrasound,^35^ and MRI,^36^ with limited success. Recent developments in dynamic CT imaging have demonstrated potential for swift diagnosis with reasonable sensitivity (~70%) and high specificity (~90%) in patients with clinical suspicion of VCD/ILO following respiratory review. CT larynx has the benefits of immediate access in many hospitals (it can be done wherever cardiac CT is done) and low radiation exposure (<1 milliSievert). If implemented early as a part of new diagnostic pathways (Figure 1), data from recent studies^37^ imply that imaging might reduce demand for laryngoscopy by up to 50% and reserve laryngoscopy for complex cases where CT larynx was negative. The latter group often need provocation including exercise testing.^37^ Overall, this approach utilizing imaging requires further research and investigation to validate implementation.

Roundtable participants emphasized the cardinal importance of case history in establishing pre‐test probability, and noted that experienced clinicians are often able to identify individuals with a higher likelihood of VCD/ILO.^38^ The overall findings on laryngoscopy determine the post‐test probability of VCD/ILO. Repeat testing is essential if the clinical suspicion (i.e., pre‐test probability) of VCD/ILO remains strong but initial laryngoscopy is negative. Follow up laryngoscopy can assess treatment response. In a small group of patients, a confirmatory diagnosis may not be achieved despite repeated testing and a pragmatic trial of laryngeal retraining may be appropriate.

Roundtable participants emphasized that swift and accurate diagnosis is essential to prevent misdiagnoses, particularly severe asthma with attendant risks of repeated oral corticosteroids, and on occasion, endotracheal intubation.^39^ Other patients may be mislabelled as ‘anaphylactic’, resulting in inappropriate adrenaline use, emergency presentations and unwarranted avoidance of suspected drugs or foods.^5^, ^6^ However, expeditious diagnosis is often not achieved, and ongoing education is essential to make health care professionals and providers aware of the condition. Advocacy for initiatives such as multidisciplinary team (MDT) clinics^40^ that promote and implement diagnostic pathways for VCD/ILO are therefore crucial to accelerate diagnosis.

## PATHOGENESIS, COMORBIDITIES AND CONTRIBUTORS

### Pathogenesis

**FIGURE 2:**
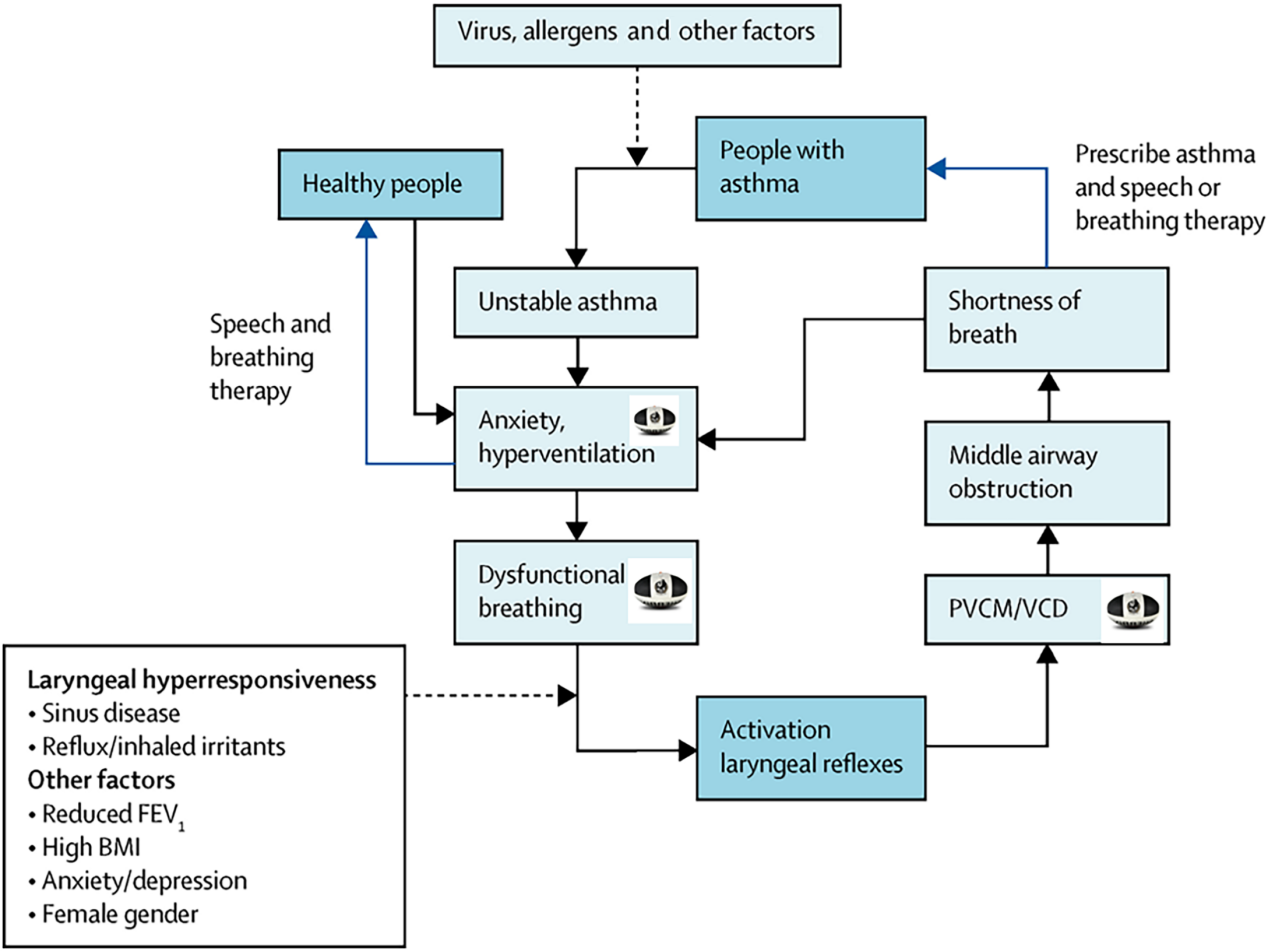
Pathogenesis. Pathogenesis model for VCD/ILO, reproduced from Bardin LRM 2017.^62^ FEV1, forced expiratory volume in 1 s; PVCM/VCD, paradoxical vocal cord motion/vocal cord dysfunction.

Understanding the pathogenesis of a condition, that is, often not recognized, variably diagnosed and the subject of very many diagnostic labels is a challenge. Patients and clinicians have accurately observed the manifestations of VCD/ILO, and these can be used to provide clues to the pathogenesis of VCD/ILO. Such clues can be obtained from symptoms and clinical characteristics of VCD/ILO and Delphi statements of agreed characteristics. Describing the pathogenesis of VCD/ILO can help explain the nature of this condition to patients and clinicians, guide management and predict outcomes. In this roundtable, these Delphi ‘clues’ were presented, along with several published mechanistic pathways (Figure 2). The participants then discussed the putative pathogenesis of VCD/ILO and associated multimorbidities.

There was consensus that VCD/ILO is a multi‐factorial, complex condition, with no single overarching pathogenic mechanism. There appear to be multiple different, possibly parallel, mechanistic pathways. This implies there may not be a single treatment, that treatment may be multifactorial and that each mechanistic pathway present may require individual treatment. This results in both complexity and heterogeneity, a challenge that has been effectively managed in other diseases in a treatable traits approach.^41^

Outside of EILO, the group identified two different types of patients, relating to the presence of two key pathogenetic elements. First, sensory hyperresponsiveness of the larynx, where laryngeal adduction is triggered by stimuli at a lower‐than‐usual threshold. Second, cortical level processes involving behavioural, functional or cortical level activation, where there has been reinforcement of behavioural input. Evidence of sensory hyperresponsiveness is demonstrated in patients who experience VCD/ILO when exposed to physical laryngeal irritants such as odour or inhaled mannitol.^42^, ^43^ On the other hand, cortical level activation appears central to VCD/ILO pathogenesis in ‘incident‐induced’ VCD/ILO, where administration of a drug, food or other putative ‘allergen’ can serve as a powerful psychogenic trigger to precipitate acute VCD/ILO,^5^, ^6^ and may also be a key factor in the prototypic ‘classic’ VCD described by Christopher.^29^ A greater understanding of these pathogenic mechanisms, the contributing factors/feeders/stimuli and the progression or possible evolution of the pathogenesis, may also explain why some people respond quickly to treatment whereas others do not. This concept has been usefully developed in chronic cough,^44^ and may be applicable to laryngeal dysfunction.^19^

The degree of response to speech pathology intervention may give insight into behavioural/cortical elements. For example, the degree of perceived empowerment by the patient and how in charge of their symptoms they feel can be predictors of speech pathology intervention success. In contrast, patients without a sense of control may not respond as well.

The group also recognized the laryngeal‐centric paradigm with the central tenet of hypersensitivity. In this, VCD/ILO is the extreme manifestation of cough, throat clearing, foreign body, globus, sensitivity and so on. Sensitivity can be central or peripheral. This equates to the ‘irritable larynx’ hypothesis proposed by Morrison.^45^

The observed clinical course of VCD/ILO can give insights to the pathogenesis. It was recognized that there can be evolution and that the condition is not static. VCD/ILO could start with exposure, but after removal of the stimulus there can be ongoing hypersensitivity. An example of this would be patients who have had an episode of true anaphylaxis and then have ongoing episodes of laryngeal closure, throat tightness and laryngeal symptoms from VCD/ILO. Subsequent episodes of VCD/ILO in such patients are often treated as anaphylaxis, though there is no evidence of anaphylaxis.^5^, ^38^ This clinical situation can be contrasted to patients who have short‐lived symptoms who have an initial exposure, such as an upper respiratory tract viral infection which triggers ongoing symptoms that lead to dysfunctional breathing and VCD/ILO, whose symptoms eventually resolve.^31^

These observations may not apply to EILO in which there is no convincing data supporting laryngeal hypersensitivity and the mechanism could be more physical in nature, related to negative‐pressure effects causing laryngeal indrawing during maximal ventilatory effort within an anatomically predisposed larynx.^14^

### Asthma, dysfunctional breathing and VCD/ILO

**FIGURE 3:**
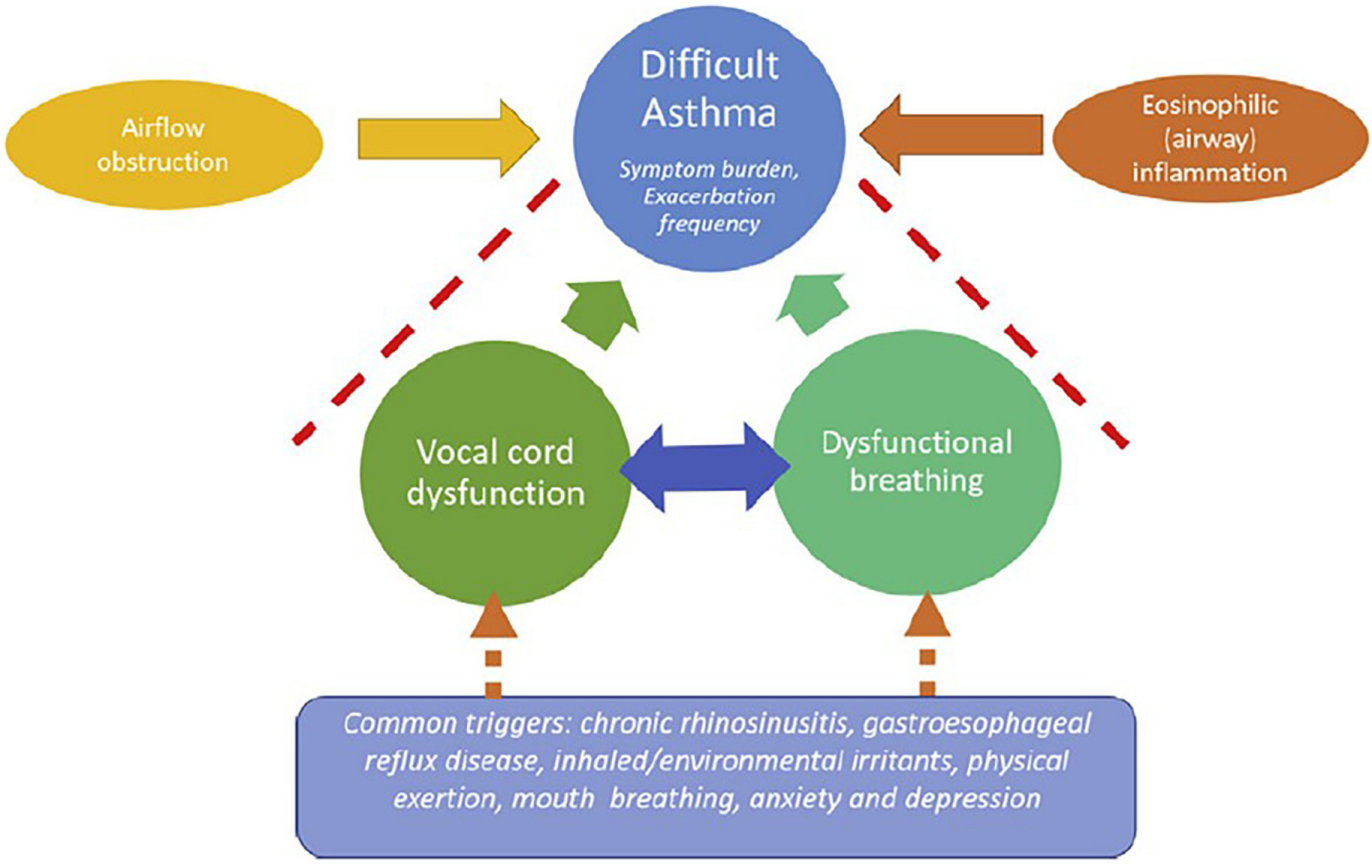
Relationship between VCD/ILO, dysfunctional breathing and difficult asthma. Reproduced from Lee et al.^30^ The relationship between VCD/ILO and its multimorbidities is complex. In this study, proposed relationships between vocal cord dysfunction, dysfunctional breathing and difficult asthma were investigated. Dashed arrow, suspected clinical associations. Dashed red line, lack of association in the study.

Asthma and dysfunctional breathing may independently cause episodic respiratory symptoms. Both may co‐exist with VCD/ILO, and interact with multimorbidities^30^, ^46^ (Figure 3). Although frequently co‐occurring, the directional contributions are unclear, specifically whether VCD/ILO leads to dysfunctional breathing or vice versa, whether there is a bi‐directional relationship, and whether treating dysfunctional breathing improves VCD/ILO. There was discussion on how respiratory muscle weakness can contribute to pathogenesis of VCD/ILO. This was exemplified with dysfunctional breathing and asthma, particularly in patients who have used chronic oral corticosteroids and may have dysfunctional breathing patterns with fixed airflow limitation. How patients with VCD/ILO respond to other intervention strategies such as management of dysfunctional breathing, or respiratory muscle training is also a relevant question.

### Comorbidities

Key multimorbidities associated with VCD/ILO include obesity, gastro‐oesophageal reflux disease, chronic rhinitis and obstructive sleep apnoea.^10^ From a mechanistic perspective, these conditions could act as triggers or precipitating factors, but they could also lead to sensitisation by upregulation of laryngeal receptors. The role of obesity as a risk factor versus comorbidity requires study. Obesity may be a strong negative predictor of speech pathology response and there were questions raised about the mechanisms, the role of dysfunctional breathing in obesity, whether the mechanisms involved are inflammatory or gastroesophageal/laryngopharyngeal reflux—or a combination. In addition, it was proposed by Roundtable participants that in the case of obesity, VCD/ILO may have greater involvement of cortical level mechanisms than local sensory elements.

## TREATMENT

**FIGURE 4:**
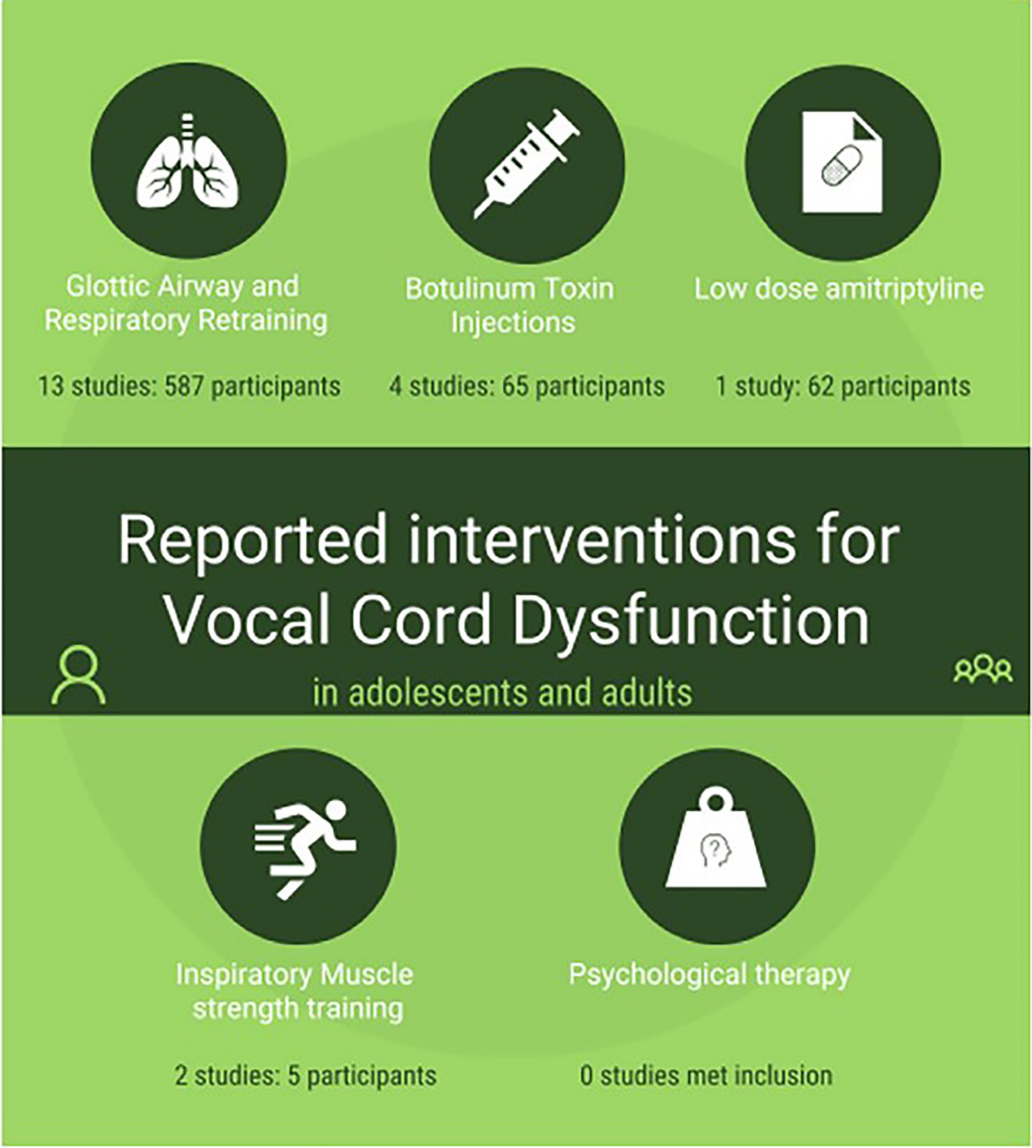
Interventions for VCD/ILO. Reproduced from Mahoney et al.^47^ Seventeen quasi‐experimental studies were found, all with high risk of bias and low evidence quality. Limited objective data exist to support effectiveness of interventions and robust controlled trials are needed.

Speech pathology is the mainstay of VCD/ILO treatment. Other interventions including botulinum toxin injection, inspiratory muscle strength training and neuromodulators (e.g., amitriptyline) have been reported.^47^ These may be used in conjunction with speech pathology treatment or when speech pathology has not been effective. There is no standard algorithm for selecting treatments or combinations. Most participants reported using speech pathology as a first‐line treatment, however, processes were variable, suggesting the need for validated clinical pathways (Figure 4).

Speech pathology management of VCD/ILO includes an initial evaluation followed by three to four therapy sessions. The evaluation includes case history, patient reported outcome measures,^48^, ^49^, ^50^, ^51^, ^52^, ^53^ voice assessment and observation of coughing and breathing at rest and during tasks. Some patients may present to speech pathologists with a confirmed diagnosis of VCD/ILO. In others, functional laryngoscopy may identify VCD/ILO when conducted either as part of the speech pathology assessment or in conjunction with otolaryngology. The evaluation should include consideration of co‐existing chronic cough, laryngeal hypersensitivity or muscle tension dysphonia as these are prevalent in VCD/ILO.^42^, ^54^

Speech pathology intervention aims to improve voluntary control over VCD/ILO by identifying the precipitating sensation and implementing laryngeal deconstriction techniques to prevent or interrupt the episode. These techniques aim to maintain abduction of the vocal folds during breathing, build up positive oral air pressure to maintain vocal fold abduction and add resistance to the exhalation. Several techniques can be employed and there is currently no evidence about their comparative effectiveness. Further key factors include addressing breath holding, increasing proficiency in exercises so that they can be recalled automatically when symptoms occur and implementation of techniques at the very first hint of a VCD/ILO episode. Patients are encouraged to note abdominal breathing and avoid large breaths when performing the exercises. Patients may need to unlearn previously taught breathing exercises from other contexts.

The specific exercises are likely to depend on patient factors and therapist preference. Speech pathologists are encouraged to keep exercises simple and introduce one at a time. The speech pathologist needs to observe the patient performing the exercise to ensure correct technique. Some patients may have tension around the laryngeal region that is preventing them from exercising optimally.

Strategies to reduce laryngeal irritation include hydration, removing laryngeal irritants and reducing phono‐traumatic vocal behaviours as well as optimizing co‐factors such as rhinitis. These strategies aim to reduce the risk of triggering a VCD/ILO episode. Desensitization may be required, especially if people believe that they are allergic to triggers.

Therapy adherence is crucial, and clinical outcomes are directly correlated to attendance. Therapy adherence factors include regular appointment attendance, regular and accurate home practice and taking initiative to implement the therapeutic strategies. The group acknowledged the need to coach patients that speech pathology as a first line treatment gives good results. Patient engagement may be influenced by who referred the patient to speech pathology. This aspect warrants further inquiry.

Education and psychoeducational counselling underpin this treatment strategy. Patients need to understand why they are implementing the strategies to optimize treatment adherence. Patient education is important, but requirements may vary. A key aspect is to internalize the understanding that the larynx can be a source of respiratory symptoms. Some individuals find it challenging to learn that symptoms may not be attributable to ‘asthma’ if they believe ‘asthma’ caused their symptoms. Research understanding the experience of patients with co‐existing asthma and VCD/ILO is required.

Participants discussed barriers to prescribing neuromodulators including amitriptyline, pregabalin and gabapentin. Some prescribers had concerns about side effects and drug–drug interactions.

Issues were raised about access to speech pathologists who had experience and confidence in managing patients with VCD/ILO. There is a need to continue upskilling speech pathologists and other healthcare professionals.

## DISCUSSION

**FIGURE 5:**
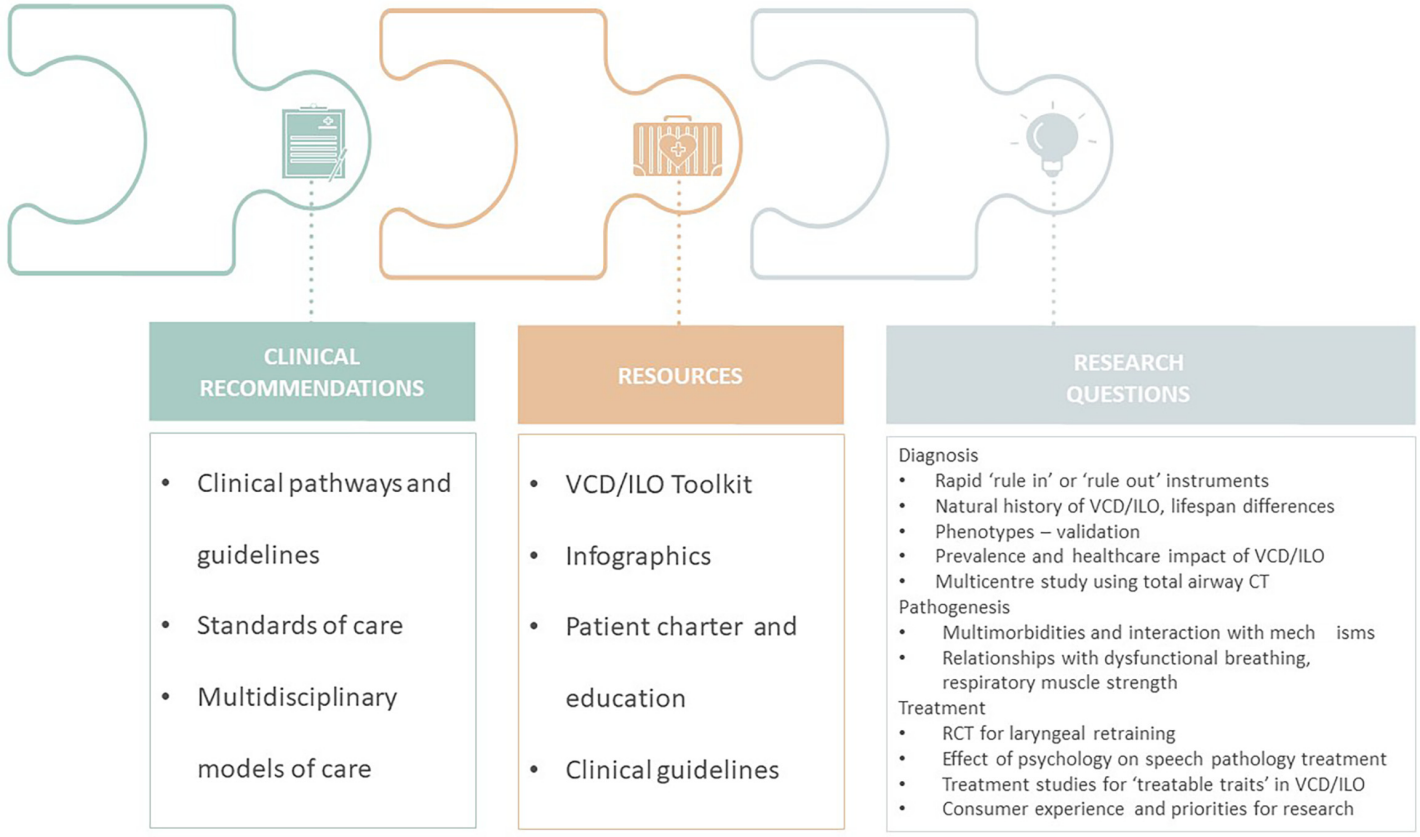
VCD/ILO Roundtable recommendations. Participants developed clinical recommendations, resource needs and research directions.

The Roundtable meeting convened a large, representative group of experienced clinicians and researchers with a track record and recognized international expertise. Following discussion of the three topics the group consolidated the ideas and summarized key recommendations that can enable progress in clinical practice, in patient outcomes and in research. Recommendations were developed in the three key areas of clinical practice, resource development and key research questions (Figure 5).

### Recommendations for clinical practice

Early diagnosis is paramount to avoid erroneous treatments that have the potential for iatrogenic consequences and increased health care burden. Improved access to diagnostic services is warranted, particularly for the gold‐standard of nasendoscopy. The group identified the need for rapid screening/testing to reduce patient burden. This was considered an important area for future clinical research and practice.

Clinical pathways are usually evidence‐based multidisciplinary care plans that aim to reduce variation in care, improve quality of care and improve outcomes for specific groups of patients.^55^ The group recommended development of clinical pathways, factoring in the individual patient and care setting. Clinicians should be able to quickly determine where to refer a patient, identify referral flags and determine what specialized tests and treatments are available.

The management of VCD/ILO benefits greatly from multidisciplinary care, however, funding for such approaches is scarce. Other disease areas such as cystic fibrosis have developed standards of care which include minimum staffing levels.^56^ Roundtable participants advocated to develop similar standards of care for VCD/ILO. However, rather than setting minimum standards for personnel, functional roles within the VCD/ILO service could be defined as a minimum standard, similar to the approach taken in the UK in severe asthma commissioning.^57^

### What are the resources we need to support this area?

Given under‐recognition, paucity of educational resources and limited clinician knowledge and skills in VCD/ILO management outside of specialty centres, participants identified the development of both patient and clinician‐focused clinical resources and education material as immediate needs. Other disease areas such as severe asthma^58^ and bronchiectasis^59^ have developed clinician toolkits, and in the Severe Asthma Toolkit (Centre of Excellence in Severe Asthma), the Vocal Cord Dysfunction section is the fifth most viewed page.

**FIGURE 6:**
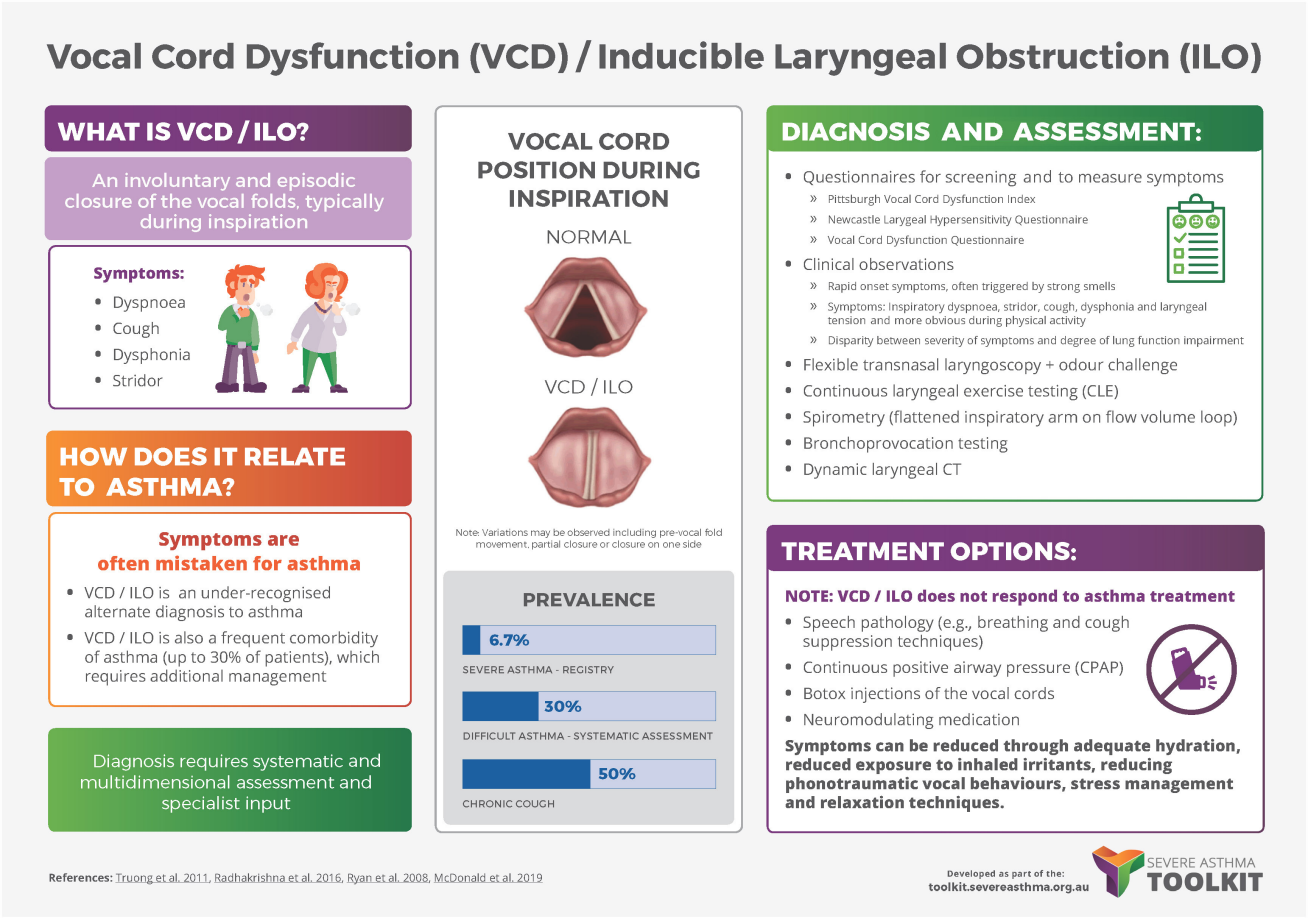
VCD infographic. Infographics portray information in a readily understandable knowledge translation format. This example is from the severe asthma toolkit https://toolkit.severeasthma.org.au/wp‐content/uploads/sites/2/2019/09/Infographic‐VCD.pdf

Roundtable participants agreed on the need for a dedicated online VCD/ILO Toolkit to support healthcare practitioners and patients, and to raise awareness of the condition and its management. The Toolkit will support identification of VCD/ILO in primary care, and trigger early referral to specialist MDT clinics. The Toolkit could include patient vignettes. Recommendations were made for specific infographics on VCD/ILO diagnostic criteria, treatments and referral pathways (Figure 6).

The needs of the consumer were recognized as a priority. Understanding the experience of the patient was an important research gap. Patient charters outline core principles that the patients should expect and have been developed in severe asthma^60^ and in COPD.^61^ A VCD/ILO patient charter was recommended to communicate what patients should expect from their care and to empower patients.

Finally, globally co‐ordinated advocacy was discussed including the possible role of a new peak society body.

### What are the research questions that need to be answered?

International multidisciplinary experts articulated numerous research questions were articulated on diagnosis, pathogenesis, treatment and patient experience. The group identified major gaps in research funding. The group advocated that funders invest in research. Specific research questions were elaborated during the Roundtable discussions. Briefly these included:

### Understanding the natural history, prevalence and impact of VCD/ILO from an international perspective

The natural history of VCD/ILO is poorly understood, particularly as it relates to age and chronicity. The group discussed the cornerstone requirement to generate multicentre data demonstrating the condition's impact (particularly healthcare and fiscal). Such a study could enhance understanding of VCD/ILO's multifaceted characteristics across continents and provide compelling rationale for diagnostic and targeted treatment studies, and aid in advocacy for resourcing.

### Multicentre study using total airway CT assessment

CT technology advances rapidly, and large decrements in radiation have been achieved which mean that dynamic laryngeal CT can be attained at doses comparable to a few months of environmental exposure. It may be possible to use CT to assess laryngeal structure and motion as part of a pathway to rule VCD/ILO in or out. CT protocols could be extended to assess sinuses and lungs during the same imaging session in selected patients, which could reveal considerable diagnostically and prognostically important information. Such approaches require investigation to determine their optimal positioning in clinical practice, and to reveal their real‐world diagnostic performance. Participants expressed interest in collaborating on a multicentre study.

### Examining the effect of laryngeal retraining on people with asthma and VCD/ILO and those with asthma and without VCD/ILO

The effect of laryngeal retraining using speech therapy needs to be established in large scale RCTs. A parallel group RCT or stepped‐wedge design was recommended to determine efficacy, and to determine the implementation potential of these and other research methodologies.

### Consumer experience and priorities for research in VCD/ILO

Major gaps were identified that relate to consumer experience of VCD/ILO and their priorities for care and research. No qualitative studies have been published to understand the needs and experiences of patients with VCD/ILO.

### Terminology

Terminology during discussions was variable. VCD, ILO, VCD/ILO and paradoxical vocal fold motion were all used, but EILO was reserved for a specific subset. Nomenclature has been the subject of considerable discussion and extensive terminology debate has an opportunity cost, possibly detracting from important clinical and research progress.

### Summary

The Roundtable explored VCD/ILO diagnosis, pathogenesis, treatment and future directions, with recommendations provided in all areas.

## AUTHOR CONTRIBUTION

**Paul Leong:** Conceptualization (equal); data curation (lead); formal analysis (equal); investigation (equal); methodology (equal); project administration (equal); writing – original draft (lead); writing – review and editing (lead). **Peter G. Gibson:** Conceptualization (equal); data curation (equal); formal analysis (equal); funding acquisition (equal); investigation (equal); project administration (equal); resources (equal); supervision (equal); validation (equal); visualization (equal); writing – original draft (equal); writing – review and editing (equal). **Anne Vertigan:** Formal analysis (equal); investigation (equal); supervision (equal); validation (equal); writing – original draft (equal); writing – review and editing (equal). **Mark Hew:** Formal analysis (equal); investigation (equal); validation (equal); visualization (equal); writing – original draft (equal); writing – review and editing (equal). **Vanessa M. McDonald:** Conceptualization (equal); formal analysis (equal); funding acquisition (equal); investigation (equal); methodology (equal); project administration (equal); supervision (equal); visualization (equal); writing – original draft (equal); writing – review and editing (equal). **Philip Bardin:** Conceptualization (equal); funding acquisition (equal); investigation (equal); methodology (equal); supervision (equal); validation (equal); visualization (equal); writing – original draft (equal); writing – review and editing (equal).

## FUNDING INFORMATION

The Roundtable was an initiative funded by the Australian National Health and Medical Research Council Centre for Research Excellence in Asthma Treatable Traits. The National Health and Medical Research had no role in study design, data collection and analysis, decision to publish or preparation of the manuscript and no industry funding was obtained. Any views expressed are those of the authors and do not necessarily represent those of the funding bodies.

## CONFLICTS OF INTEREST STATEMENT

Mark Hew declares consulting fees to GlaxoSmithKline, AstraZeneca, Novartis and Sanofi as well as honoraria for GlaxoSmithKline, AstraZeneca, Novartis, Sanofi, Chiesi and Teva all paid to his employer. Philip G. Bardin and Vanessa M. McDonald are Editorial Board members of Respirology and a co‐author of this article. They were excluded from all editorial decision‐making related to the acceptance of this article for publication.

## Supporting information


**Appendix S1:** Supporting Information.

**Appendix S2:** Supporting Information.
