# Supplementary material for: Vocal cord dysfunction/inducible laryngeal obstruction—2022 Melbourne Roundtable Report
Source: Respirology. 2023 May 23;28(7):615–26. doi: 10.1111/resp.14518 (PMC10947219; doi:10.1111/resp.14518)
Supplement: Supplementary file 1 — Appendix S1: Supporting Information. [file RESP-28-615-s002.docx]

**Supporting Information S1**

**Roundtable Supporting Information**

The Roundtable was an activity of The Australian National Health and Medical Research Council Centre of Excellence in Asthma Treatable Traits. The Roundtable Steering Committee was comprised of the authors of this paper.

Four topics were identified for discussion and hence there were four sessions. These were diagnosis, pathogenesis, treatment, and future directions. The respective goals for the sessions were to explore pathogenesis and contributors, outline management and model(s) of care, and delineate research questions.

Attendees were identified by the Steering Committee, comprised of the authors of this paper and attendance was coordinated by the Centre of Excellence in Asthma Treatable Traits. We aimed to include a diverse range of attendees regarding gender, age, geography and craft group. All had been identified as having an interest in the topic from a clinical or research perspective.

Each session ran for 90 minutes. Sessions commenced with a 15-minute presentation to prompt discussion followed by 30 minute break out sessions, and then 45 minutes for group discussion. No formal literature review was conducted but presenters had all published on their specific topic, and attendees were generally versed in the field.
