# Supplementary material for: Vocal cord dysfunction/inducible laryngeal obstruction—2022 Melbourne Roundtable Report
Source: Respirology. 2023 May 23;28(7):615–26. doi: 10.1111/resp.14518 (PMC10947219; doi:10.1111/resp.14518)
Supplement: Supplementary file 2 — Appendix S2: Supporting Information. [file RESP-28-615-s001.docx]

**Supporting Information S2 – Roundtable Attendees**

Professor Vanessa McDonald, Professor of Nursing, Centre of Excellence in Asthma Treatable Traits, University of Newcastle, Newcastle, NSW, Australia

Conjoint Professor Peter Gibson, Respiratory Physician, Centre of Excellence in Asthma Treatable Traits, University of Newcastle and Hunter New England Health, John Hunter Hospital, Newcastle, NSW, Australia

Professor Philip Bardin, Respiratory Physician, Monash University, Melbourne, VIC, Australia

Dr Paul Leong, Respiratory & Sleep Physician, Monash Health, Monash University, Melbourne, VIC, Australia

Dr Vanessa Clark, Researcher, Centre of Excellence in Asthma Treatable Traits, University of Newcastle, Newcastle, NSW, Australia

Dr Rebecca McLoughlin, Researcher, Centre of Excellence in Asthma Treatable Traits, University of Newcastle, Newcastle, NSW, Australia

Professor Mark Hew, Respiratory Physician and Immunologist, Alfred Health and Monash University, Melbourne, VIC, Australia

Conjoint Associate Professor Anne Vertigan, Speech Pathology, University of Newcastle and Hunter New England Health, John Hunter Hospital, Newcastle, NSW, Australia

Associate Professor Janet Rimmer, Respiratory Physician, Woolcock Institute of Medical Research, University of Sydney and St Vincents Clinic, Sydney, NSW, Australia

Dr Janine Mahony, Speech Pathologist, Alfred Health, Latrobe University, Melbourne, VIC, Australia

Emeritus Professor Jennifer Oates, Speech Pathologist, La Trobe University, Melbourne, VIC, Australia

Mr Malcolm Baxter, ENT Surgeon, Monash Health, Monash University, Melbourne, VIC, Australia

Associate Professor Debbie Phyland, Speech Pathologist, Monash Health, Monash University, Melbourne, VIC, Australia

Professor John Upham, Respiratory Physician, Prince Alfred Hospital, University of Queensland, Brisbane, QLD, Australia

Ms Adriana Avram, Nurse, Monash Health, Monash University, Melbourne, VIC, Australia

Dr Kathy Low Respiratory and Sleep Physician Monash Health, Monash University, Melbourne, VIC, Australia

Dr Joy Lee, Respiratory & Sleep Physician, Alfred Hospital, Monash University, Melbourne, VIC, Australia

Dr Stephanie Stojanovic, Respiratory & Sleep Physician, Alfred Hospital, Monash University, Melbourne, VIC, Australia

Professor James Hull, Respiratory Physician, Royal Brompton Hospital, London, United Kingdom

Professor Vibeke Backer, Respiratory Physician, Rigshospitalet Copenhagen University Hospital, University of Copenhagen, Denmark

Associate Professor Andrej Petrov, Allergist-Immunologist, University of Pittsburgh, Pennsylvania, United States of America

Dr Tom Carroll, ENT surgeon, Department of Otolaryngology-Head and Neck Surgery, Harvard Medical School, Division of Otolaryngology-Head and Neck Surgery, Brigham and Women’s Hospital

Dr Elaine Yap, Respiratory Physician, Department of Respiratory Medicine, Te Whatu Ora Counties, Middlemore Hospital, Auckland, New Zealand

Ms Elizabeth Leahy, Nurse, Monash Health, Melbourne, VIC, Australia

Mr Anthony Flynn, Asthma Australia, Melbourne, VIC, Australia and University of Newcastle, NSW Australia

Professor Thomas Halvorsen, Paediatric Respiratory Consultant, Department of Paediatric and Adolescent Medicine, Haukeland University Hospital, Bergen, Norway, Faculty of Medicine, Department of Clinical Science, University of Bergen, Bergen, Norway.

Dr Li Ping Chung, Respiratory Physician, Fiona Stanley Hospital, Perth, University of Western Australia, WA, Australia

Associate Professor Hege Clemm, Paediatric Respiratory Consultant, Haukeland University Hospital, University of Bergen, Bergen, Norway, Department of Paediatric and Adolescent Medicine, Haukeland University Hospital, Bergen, Norway, Faculty of Medicine, Department of Clinical Science, University of Bergen, Bergen, Norway.

Ms Cathy Prendergrast, Respiratory Physiologist & Medical Scientist, Women’s and Children’s Hospital Adelaide, University of South Australia, Australia

Ms Claire Slinger, Consultant Respiratory Speech And Language Therapist, Lancashire Teaching Hospitals Trust, England, United Kingdom

Dr Joo Koh, ENT, Monash Health, Monash University, Melbourne, VIC, Australia
